# A Next‐Generation ELISA for the Detection of Anti‐(Para)Nodal Antibodies in Autoimmune Nodopathy and COVID‐19 Vaccinated Individuals

**DOI:** 10.1111/jns.70117

**Published:** 2026-03-29

**Authors:** Luise Appeltshauser, Chiara Ruprecht, Julia Reusch, Juliane Mees, Helena Glenewinkel, Claudia Sommer, Manuel Krone, Isabell Wagenhäuser, Kathrin Doppler, Nils Petri

**Affiliations:** ^1^ Department of Neurology University Hospital Würzburg Würzburg Germany; ^2^ German Center for Neurodegenerative Diseases (DZNE) Berlin Berlin Germany; ^3^ Department of General, Visceral, Transplantation, Vascular and Pediatric Surgery University Hospital Würzburg Würzburg Germany; ^4^ Central Laboratory Unit University Hospital Würzburg Würzburg Germany; ^5^ Department of Anesthesiology, Intensive Care, Emergency Medicine and Pain Medicine, Centre for Interdisciplinary Pain Medicine University Hospital Würzburg Würzburg Germany; ^6^ Department of Pediatrics University Hospital Würzburg Würzburg Germany; ^7^ Department of Internal Medicine I University Hospital Würzburg Würzburg Germany

**Keywords:** autoimmune nodopathy, contactin‐1, COVID‐19, ELISA, neurofascin, SARS‐CoV‐2

## Abstract

**Background and Aims:**

Autoimmune nodopathy (AN) is a subtype of antibody‐mediated inflammatory neuropathy targeting the node of Ranvier (NoR). Diagnosis requires detection of anti‐(para)nodal autoantibodies like contactin‐1 and neurofascin‐155 via ELISA or cell‐based assays, but protocols are inconsistent. Causes of node autoimmunity are unknown, and respiratory infections, including SARS‐CoV‐2 infection or COVID‐19 vaccination as triggers, have not been thoroughly investigated. We aim to establish and validate a next‐generation automated ELISA for anti‐(para)nodal antibodies and investigate whether low‐titer antibodies occur in recently COVID‐19‐vaccinated healthy individuals.

**Methods:**

We used the Ella platform to customize an automated ELISA for anti‐contactin‐1, −neurofascin‐155, and ‐Caspr‐1 serum IgG. Patients with known AN (23 anti‐neurofascin, 13 anti‐contactin‐1, and 8 anti‐Caspr‐1), 64 patients with seronegative (sub) acute inflammatory neuropathies, and 30 healthy controls served for validation, including quality analysis versus standard ELISA. Thirty‐seven diagnostic samples of patients with suspected AN and 280 sera of healthcare workers included in the CoVacSer study, collected 3 weeks after COVID‐19 vaccination or infection, were tested for anti‐contactin‐1 and anti‐neurofascin‐155.

**Results:**

The automated ELISA showed high sensitivity (87.5%–100%) and specificity (98.4%–100%) for identifying AN, with reduced hands‐on time, high automation, and similar quality and titer characteristics as standard ELISA. Low‐titer anti‐neurofascin‐155, but no anti‐contactin‐1 autoantibodies, were detected in 1/280 post‐SARS‐CoV‐2‐vaccination or infection sera (0.36%). Longitudinal testing and clinical assessment did not indicate SARS‐CoV‐2‐related neurological symptoms.

**Interpretation:**

We provide a highly automated, rapid, universally applicable test platform for anti‐(para)nodal antibodies. The low frequency of anti‐(para)nodal antibodies and absence of clinical AN manifestations in COVID‐19‐vaccinated individuals support vaccination safety regarding AN development.

## 1 Introduction

Autoimmune nodopathy (AN) represents a distinct and recently recognized immune‐mediated peripheral nerve disorder, characterized by pathogenic autoantibodies targeting nodal and paranodal adhesion molecules such as contactin‐1, neurofascin‐155, and Caspr‐1 [1, 2]. The autoimmune attack disrupts the integrity of the node of Ranvier (NoR), impairing saltatory conduction and resulting in motor and sensory deficits that often resemble Guillain‐Barré syndrome (GBS), or chronic inflammatory demyelinating polyradiculoneuropathy (CIDP). Distinct clinical features frequently include a (sub)acute onset, tremor, pronounced ataxia, early age of manifestation, cranial nerve involvement, and severe course, sometimes including respiratory failure [1, 2]. Some patients also develop neuropathic pain or concurrent nephropathy [3, 4].

While the precise triggers of autoimmunity against the NoR remain unclear, HLA II‐dependent antigen‐presenting T‐helper cells might be involved, and predisposing factors like type 2 diabetes have been identified [2, 5]. GBS is frequently preceded by infections such as *Campylobacter jejuni* , influenza, Zika virus, and, in some cases, by SARS‐CoV‐2, and can also be associated with vaccinations [6, 7, 8]. In acute‐onset AN, antecedent gastrointestinal or respiratory infections are also reported in up to 44% of patients [3, 9, 10]. Single cases with anti‐(para)nodal antibodies in COVID‐19 and postvaccination‐related acute neuropathy have been reported [11, 12, 13]. The paranodal adhesion protein contactin‐1 has been identified as a specific binding partner of the SARS‐CoV‐2 receptor binding domain, which may potentiate viral infection, and is upregulated in COVID‐19 patients [14], but a direct pathogenetic link between SARS‐CoV‐2 infection/vaccination and the development of GBS or even AN is still missing [7]. To date, it is unclear if SARS‐CoV‐2 infection or COVID‐19 vaccination may induce anti‐(para)nodal antibody responses in healthy individuals.

Accurate and timely diagnosis of AN is critical as it directly impacts treatment strategies and prognosis. Patients respond poorly to standard treatment like intravenous immunoglobulins but show a good response to B‐cell depleting therapy and could benefit from early and aggressive treatment [1, 10, 15, 16, 17]. Anti‐(para)nodal antibodies can be detected using ELISA, immuno‐DOT, cell‐based assays (CBA), and binding assays on teased nerve fibers, with the highest accuracy when using live CBA [1, 18, 19, 20]. Still, these assays are time‐consuming, not ubiquitously available, and need a high level of expertise for analysis [21]. Overall interlaboratory concordance for positive results is only 75% in established testing centers, with poor concordance regarding subclass‐specificity [18]. These challenges underscore the need for improved diagnostic tools, including automated assays capable of high‐throughput screening, enabling easy and precise diagnosis and monitoring of AN in large patient cohorts.

Recent advances in assay technology have introduced platforms such as the Ella system, which employs microfluidics‐based automation for immunoassay development [22]. It can be applied for the detection of biomarkers in human samples and reaches equivalent results to established ultrasensitive reference methods [23, 24, 25]. Further, it allows multiplexing, and its customizable cartridge design allows for the application in new biomarker and antibody studies [26]. This next‐generation ELISA offers advantages over traditional methods, such as increased assay speed, automation, and high sensitivity, thus potentially overcoming current diagnostic limitations.

In this study, we aim to develop and validate a highly automated, high‐throughput next‐generation ELISA platform using the Ella system to investigate whether anti‐(para)nodal autoantibodies can be detected with improved accuracy and scalability. Applying the new method, we seek to explore whether low‐titer anti‐(para)nodal antibodies can be detected in a large cohort of individuals after mRNA‐based COVID‐19 vaccination or SARS‐CoV‐2 infection.

## 2 Materials and Methods

### 2.1 Patients and Study Cohort

Serum samples with antibodies against contactin‐1 (*n* = 13), Caspr‐1 (*n* = 8), neurofascin‐155 (*n* = 11), or pan‐neurofascin (*n* = 12) were identified from patients who underwent diagnostic testing at the Department of Neurology, University Hospital Würzburg, Germany, between 2015 and 2023 as previously described [10, 27, 28, 29, 30]. Inclusion criteria for patients with the definite diagnosis of AN (“seropositive”) were positive results for anti‐(para)nodal antibodies in the teased fibers and ELISA screening assay, and fixed CBA for confirmation, among clinical and electrophysiological findings typical for AN. Sixty‐four sera from sex and age‐matched patients diagnosed with acute‐onset CIDP tested negative for anti‐(para)nodal antibodies served as a control group (“seronegative”). Thirty sera from individuals without autoimmune, malignant, or neurological conditions recruited for previous studies served as healthy controls [30, 31]. As a prospective test cohort, we included all samples with suspected AN that were referred to our center for diagnostic testing from 1st of January 2024 to 31st of December 2024. Inclusion criteria for this test cohort were all samples from adult patients (> 18 years) with typical AN features, namely an acute to subacute onset, and motor predominant neuropathy with an inability to walk freely. This resulted in a test cohort of *n* = 37 samples, which were screened for anti‐contactin‐1 and anti‐neurofascin‐155 using the Ella platform by a blinded researcher. Results were then compared to standard ELISA and CBA results.

Further, we included sera of 280 SARS‐CoV‐2 vaccinated individuals participating in the *CoVacSer* Study at University Hospital Würzburg. The *CoVacSer* study is a prospective, longitudinal cohort study examining long‐term Anti‐SARS‐CoV‐2 immunity and its determinants after SARS‐CoV‐2 infection and/or COVID‐19 vaccination in > 1800 healthcare workers during the COVID‐19 pandemic (29th of September 2021 to 31st of December 2023) [32, 33, 34]. Serum samples taken with a mean interval of 22 (2–100) days from the last SARS‐CoV‐2 infection or COVID‐19 vaccination (only mRNA‐based and EMA‐approved vaccines considered) were included (see Table S1), falling within the range of peak autoantibody levels in AN‐related diseases such as GBS [35]. Anti‐SARS‐CoV‐2 spike IgG was determined using the SERION ELISA *agile* SARS‐CoV‐2 IgG (SERION Diagnostics, Würzburg, Germany). In neuropathy patients and healthy controls, clinical data, including total IgG levels (available in *n* = 20 participants), were assessed retrospectively from patient charts and previous studies [28], whereas data from CoVacSer participants were assessed prospectively. Descriptive cohort data are shown in Table S1. The study was approved by the Ethics Committee of the University of Würzburg following the Declaration of Helsinki (reference numbers 220/20 and 79/21), and participants had given written informed consent.

### 2.2 Digoxigenin Conjugation

Human contactin‐1 (#10383‐H08H, Sino Biological, Beijing, China), Caspr‐1 (#2418‐CR, Bio‐Techne, Minneapolis, Minnesota, USA) neurofascin‐155 [36], neurofascin‐186 [36] and bovine serum albumin (BSA, #A2153, Merck KGaA, Darmstadt, Germany) were conjugated to digoxigenin according to the instructions of the manufacturer (Simple Plex 48‐Digoxigenin Cartridge Quick Start Guide). In brief, Digoxigenin NHS‐ester (#ENZ‐45022, Enzo Life Sciences, Lausen, Switzerland) was reconstituted at 0.67 mg/mL in N, *N*‐dimethylformamide (DMF, #270547, Merck KGaA). For conjugation, 150 μL of the respective protein (reconstituted 1 mg/mL in PBS) was mixed with a 5‐fold molecular excess of digoxigenin and 16.8 μL of sodium bicarbonate (reconstituted 75 mg/mL in DI, #S8875, Merck) and incubated for 1 h in the dark. To calculate the volume of needed digoxigenin, the following formula was applied:

$$\begin{array}{cc} \text{Volume}\left(\mathrm{DIG}\;\left[\mathrm{mL}\right]\right)=\; & \frac{0.15\;\mathrm{mg}\;\left(\text{protein mass}\right)}{\text{protein molecular weight}\;\left[\frac{g}{\mathrm{mol}}\right]}\\ & *5\;\left(\mathrm{DIG}\;\text{molar excess}\right)\\ & *\frac{658\frac{g}{\mathrm{mol}\;}\left(\mathrm{DIG}\;\text{molecular weight}\right)}{0.67\frac{\mathrm{mg}}{\mathrm{mL}}\;\left(\mathrm{DIG}\;\text{solution concentration}\right)} \end{array}$$

The free label was removed using Zeba Spin Desalting Columns, 40 K MWCO (#A57759, Thermo Fisher Scientific, Waltham, Massachusetts, USA) according to the manufacturer's instructions. Conjugated protein was aliquoted and stored at −20°C until use. We evaluated the success of digoxigenin‐conjugation using an anti‐neurofascin‐155, anti‐neurofascin‐186, anti‐contactin‐1, and anti‐Caspr‐1 ELISA as previously described [27, 29, 30], but with the following modifications: coating in duplets was performed with PBS only as a negative control, with the original protein and digoxigenin‐conjugated protein. Proteins were diluted at 2 mg/mL (contactin‐1 and Caspr‐1 ELISA) or 5 mg/mL (neurofasin‐155 and ‐186 ELISA). Commercial protein‐specific antibodies, a healthy control serum, and seropositive patient serum with the respective known antibody (anti‐contactin‐1, anti‐Caspr‐1, or anti‐neurofascin‐155/186) served as primary antibodies. Secondary antibody incubation included HRP‐conjugated anti‐digoxigenin antibody (0.1 μg/mL, #ab51949, Abcam, Cambridge, U.K.), and anti‐human, anti‐chicken, anti‐mouse, and anti‐rabbit IgG HRP‐conjugated secondary antibodies as previously described [27, 29, 30].

### 2.3 Ella Customization

We used SimplePlex 48‐Digoxigenin Cartridges (#952927, Bio‐Techne) for the Ella platform (Bio‐Techne) to customize an automated next‐generation ELISA, following a modified protocol from the manufacturer [26]. We titrated optimal dilutions for the diluents, capture proteins, sample, and detection antibody, defined as high relative fluorescence unit (RFU) values for positive controls and low values for healthy control and background control (without capture protein, sample, or detection antibody). Digoxigenin‐conjugated capture proteins (BSA as background control, contactin‐1, neurofascin‐155, neurofascin‐186, Caspr‐1) were diluted from 1 to 15 μg/mL in Reagent Diluent included in the cartridge package (#PN 895182, Bio‐Techne) or different blocking solutions containing up to 50% EX‐CELL CD CHO Serum‐Free Medium (#14360C, Merck) and 5% fetal bovine serum (#26140079, Thermo Fisher Scientific). Samples used for titration included commercial antibodies against contactin‐1, pan‐neurofascin, or Caspr‐1; three healthy control sera; and two seropositive patients with known AN per target antigen, with low (1:100–1:500) and high (1:15000–1:30000) titers, respectively. Human serum samples were titrated from 1:10 to 1:10000. Different commercial antibodies against contactin‐1 (#ab191285, #ab66265, #ab105582 Abcam; #AF904, Bio‐Techne), neurofascin (#AF3235, Bio‐Techne; #ab31457, Abcam; #15035 Cell Signaling Technology, Danvers, MA, USA), and Caspr‐1 (#Sc‐374 489 and #Sc‐373 777, Santa Cruz Biotechnology, Dallas, USA; #ab34151, Abcam) were tested at different dilutions. Six sample diluents (SD 06, SD 10, SD 13, SD 19, SD 20, SD 42) from two Ancillary diluent packs (#992–522 and #992–523, Bio‐Techne) and ELISA sample diluent [27] were evaluated. Different biotinylated detection antibodies directed against human and anti‐host IgG, depending on the commercial antibody used (#GtxHu‐004‐F2BIO, #RbxGt‐004‐EBIO, #GtxCk‐003‐DBIO, ImmunoReagents, Raleigh, NC, USA; #BA‐1000‐1.5, #BA‐9500‐1.5, BA‐9010‐1.5, #BA‐9200‐1.5, #BA‐2001‐1.5, VectorLabs, Newark, CA, USA) were tested diluted from 0.075 to 5 μg/mL.

We evaluated the use of serum‐specific background normalization by using digoxigenin‐conjugated BSA as an alternative capture protein for every human sample. Substances and concentrations for the final protocol are listed in Table 1. Preparation of substances, pipetting onto the Ella cartridge at a volume of 50 μL per sample, and Ella run were performed according to the manufacturer's instructions [26]. For analysis, the mean RFU from three independent GNRs per sample was calculated, discarding single results with coefficients of variation (CV) > 10%.

**TABLE 1:** Protocol for a next‐generation ELISA for the detection of anti‐(para)nodal antibodies.

	Contactin‐1	Neurofascin‐155	Caspr‐1	Neurofascin‐186
Layout 48‐digoxigenin cartridge (#952927, bio‐techne)	One background control (without sample) One positive control (commercial or AN serum) *n* = 46 samples/cartridge Prepare diluted sample aliquots on ice			
Serum‐specific background normalization	If mean sample RFU is above threshold, repeat including two wells/sample (one capture target protein, one with digoxigenin‐labeled capture BSA, Merck #A2153, at the same dilution) and substract mean RFU of BSA, using the threshold of substracted RFU of healthy controls.			
Digoxigenation	Digoxigenin NHS‐ester (#ENZ‐45022, enzo life sciences)			
Capture				
Protein	Recombinant human contactin‐1 protein (sino biological, #10383‐H08H)	Full‐length human neurofascin‐155 [36]	Recombinant Human Caspr1 protein (biotechne, # 2418‐CR)	Full‐length human neurofascin‐186 [36]
Concentration	10 μg/mL	10 μg/ml	2 μg/ml	5 μg/ml
Diluent	Reagent diluent			
Additional blocking	None			
Sample				
Commercial ab	None (use AN serum as positive control)	Chicken anti‐pan‐neurofascin IgY 1:1000 (biotechne, #AF3235)	Mouse anti‐Caspr‐1 IgG 1:1000 (Santa Cruz biotechnology Inc., #Sc‐373 777 (E‐8))	Chicken anti‐pan‐neurofascin IgY 1:1000 (biotechne, #AF3235)
Human serum	1:50	1:100	1:100	1:100
Sample diluent	SD 06			
Detection ab				
Human sample	Anti‐human IgG (ImmunoReagents, #GtxHu‐004‐F2BIO)			
Dilution	5 μg/ml	2 μg/ml	2 μg/ml	2 μg/ml
Commercial positive control	None	Anti‐chicken IgG 2 μg/ml (VectorLabs #BA‐9010‐1.5)	Anti‐mouse IgG 3.5 μg/ml (VectorLabs #BA‐9200‐1.5)	Anti‐chicken IgG 2 μg/mL (VectorLabs #BA‐9010‐1.5)
Diluent	Reagent diluent			
Pipetting and evaluation	50 μl per capture protein, sample, and detection ab Add 1 mL of wash buffer to reservoirs Run according to manufacturer's instructions Evaluate mean RFU of three GNR Discard values with CV of > 10%			

Abbreviations: Ab = antibody, BSA = bovine serum albumin, GNR = glass nanoreactor, RFU = relative fluorescence units.

### 2.4 Ella Performance and Quality Assessment

The customized final protocol (see Table 1) was used to determine thresholds for raw positive results, which were defined as RFU values above > 3 standard deviations (SD) of the mean of *n* = 30 healthy control sera for each target antigen (except *n* = 10 healthy control sera for neurofascin‐186). Furthermore, thresholds for serum‐specific background normalization were determined by using two wells per control serum (one with the target protein and one with BSA capture) and subtracting the respective mean RFU. Cohort samples were then analyzed subsequently, running two cartridges per test day (*n* = 92 samples/day) and measuring hands‐on time. In case of mean RFU values above the threshold, the assay was repeated independently, including serum‐specific background normalization (see Table 1), and only considered positive if results were above the normalized threshold.

Results for antibodies against Caspr‐1, Contactin‐1, and Neurofascin‐155 of patients with AN and seronegative immune‐mediated neuropathies were used to determine diagnostic sensitivity and specificity compared to the in‐house gold standard diagnostic assays (typical clinical phenotype, positive in standard ELISA, and confirmed by CBA). Titer assessment was performed exemplarily for three AN patients per target antigen using a dilution series, defining the titer as the last dilution above the threshold. Intra‐assay variability was examined by repeating three healthy control sera and three AN patient samples in ten different wells within one cartridge and assessing CVs. Inter‐assay CVs were calculated by repeating these six samples on five independent cartridges and test days. Quality assessment was not performed for anti‐neurofascin‐186. Here, due to limited cartridge space, only a reduced cohort of *n* = 10 healthy controls, *n* = 6 patients with seronegative neuropathy, *n* = 3 patients with anti‐pan‐neurofascin related AN and *n* = 1 patient with anti‐neurofascin‐186 antibodies were tested.

Thirty‐seven patients from a prospective test cohort of suspected AN (see above) and 280 SARS‐CoV‐2 vaccinated individuals were tested for anti‐contactin‐1 and anti‐neurofascin‐155 antibodies. The antibody titer in patients with newly detected antibodies was determined as described above, and longitudinal analysis with follow‐up samples (*n* = 4) was performed in one positive case. Runs were only considered for analysis if negative and positive controls worked (which was always the case).

### 2.5 Standard ELISA and Immunofluorescence Binding Assays

All patient samples with AN and inflammatory neuropathy had been assessed via customized standard ELISA and teased fiber binding assay as previously described [27, 29, 30] during routine diagnostic testing. Positive samples were validated using CBA [3, 27, 29]. Samples positive in the new next‐generation ELISA (including the cohort of SARS‐CoV‐2 vaccinated individuals) were repeated with standard ELISA, teased fibers binding assay, and CBA for validation, including colocalization studies with human IgG and chicken anti‐pan‐neurofascin IgY antibody (AF3235, R&D Systems Inc., Minneapolis, U.S.) and corresponding Cy3‐ and AlexaFluor 488‐labeled secondary antibodies (JacksonImmunoResearch, Westgrove, Pennsylvania, U.S.). Intra‐assay and inter‐assay CVs were determined via standard ELISA as described above, and mean CVs were compared to Ella quality assessment. Two ELISA plates were assessed per test day, with *n* = 22 samples/plate, and hands‐on time was measured per test day. Sample and protein volumes needed for testing were compared between Ella and ELISA.

### 2.6 Statistics and Software

Simple Plex Runner 4.0.0.28 for Windows 10 (Bio‐Techne) was used for the Ella run, and Ella and standard ELISA raw data were saved and analyzed in Simple Plex Explorer (Bio‐Techne) and Microsoft Excel 365 MSO (Version 2503). Receiver Operating Characteristic (ROC) curves were calculated using the Wilson/Brown method, with DeLong test for comparing the Area Under the Curves (AUC) to the random classifier. Inter‐assay and intra‐assay CV for Ella and standard ELISA, as well as threshold values, were compared using Wilcoxon test or paired *t*‐test, depending on the normal distribution of values per target antigen. Spearman or Pearson correlation of RFU versus OD values for the test cohort, Ella and ELISA titer correlation, total IgG levels vs. anti‐SARS‐CoV‐2 spike IgG levels, and mean RFU values were calculated. *p* values < 0.05 were considered statistically significant. GraphPad Prism Version 10.0.0 (GraphPad Software, Boston, Massachusetts, USA) was used for statistical testing and display. Further, Biorender.com and Adobe Illustrator (Adobe Inc., San José, CA, US) were used for figure design.

## 3 Results

### 3.1 A Fast and Automated Next‐Generation ELISA for the Detection of Anti‐(Para)Nodal Antibodies

**FIGURE 1:**
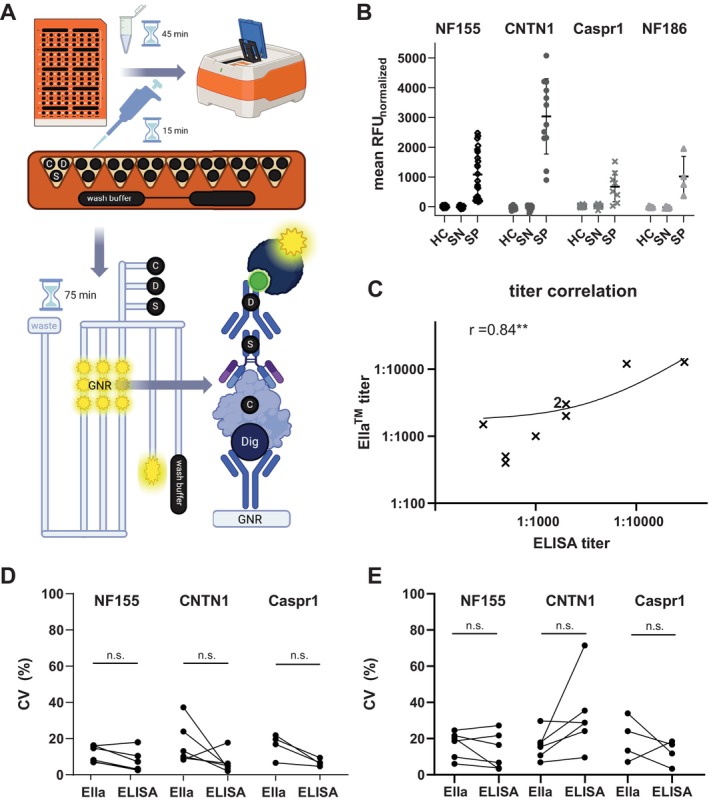
Ella for the detection of anti‐(para)nodal antibodies. (A) Schematic display of the Ella procedure, including hands‐on time for sample dilution and pipetting, and hands‐free time during the automated Ella run. Abbreviations: C = capture protein, D = detection antibody, GNR = glass nanoreactor, S = sample (human serum). Created with BioRender.com (B) Mean RFU values after serum‐specific background normalization in healthy controls (HC), seronegative (SN) and seropositive (SP) patients, using neurofascin‐155 (NF155), contactin‐1 (CNTN1), Caspr‐1, and neurofascin‐186 (NF186) as target proteins. (C) Spearman correlation of serum titers of *n* = 9 AN patients by Ella (*y*‐axis) and ELISA (*x*‐axis) on a logarithmic scale. “2 × ” represents two different patient samples with identical titers (1:2000 in both assays). The line shows the linear regression curve. **p ≤ 0.01 (D) Intra‐Assay coefficient of variation (CV) in six samples, compared between Ella and ELISA. N.s. = not significant. (E) Inter‐Assay CV in six samples, compared between Ella and ELISA. N.s. = not significant.

Digoxigenin conjugation of all target proteins was successful, as validated by ELISA (see Figure S1). Using a digoxigenin‐labeled customizable 48‐well cartridge, we established a customized protocol for an automated next‐generation ELISA for the Ella platform that reliably detected antibodies against contactin‐1, Caspr‐1, neurofascin‐155 and neurofascin‐186 in human serum, as summarized in Table 1 and Figure 1A. Commercial positive controls were successfully established for Caspr‐1 and neurofascin‐155/186, whereas all tested anti‐contactin‐1 antibodies produced negative results. The automated Ella workflow reduced hands‐on time per sample from 6.8 to 0.6 min/sample compared to standard ELISA. The sample volume needed per probe was reduced by 50%–75% (4 μL/sample for ELISA vs. 1–2 μL/sample for Ella), depending on the target antigen. Protein volume was reduced by 50% for neurofascin‐155 (1 μg/sample vs. 0.5 μg/sample), by 75% for neurofascin‐186 (1 μg/sample vs. 0.25 μg/sample), by 75% for Caspr‐1 (0.4 μg/sample vs. 0.1 μg/sample), and increased by 25% for Contactin‐1 (0.4 μg/sample vs. 0.5 μg/sample).

### 3.2 Serum‐Specific Background Normalization Increases Assay Sensitivity and Specificity

We determined the mean RFU values of healthy controls, seropositive, and seronegative patients for antibodies against contactin‐1, Caspr‐1, and neurofascin‐155/186. Values did not correlate with total IgG levels in all groups (*p *> 0.05). Thresholds and positive results for the respective target antigen are shown in Table 2 and Figure S2. Diagnostic test sensitivity ranged from 75% to 100%, and specificity from 85.9% to 100%. Next, we determined normalized thresholds by including serum‐specific background normalization, thus subtracting the mean RFU when using digoxigenin‐labeled BSA as a capture protein complementary to the target protein. Mean RFU values were strongly reduced (*p* < 0.0001 for all target antigens), and the diagnostic sensitivity and specificity of the assay could be strongly increased (87.5%–100% and 98.4%–100%, respectively). Test accuracy was now 100% (for anti‐contactin‐1 and anti‐neurofascin‐155/186) and 97.2% for anti‐Caspr‐1 antibodies. Normalized mean RFU values are shown in Figure 1B, ROC analysis, including AUCs, is shown in Figure S2. Thus, serum‐specific background normalization enhances assay performance, achieving sensitivity and specificity levels equivalent to standard ELISA and CBA [18].

**TABLE 2:** Threshold values for positive results in the next‐generation ELISA.

	Contactin‐1	Caspr‐1	Neurofascin‐155	Neurofasicn‐186^###^
Raw values Ella				
Threshold (RFU)	227.4	196.6	176.8	158.6
Seronegative cohort	9/64	8/64	9/64	0/6
Seropositive cohort	13/13	6/8	23/23	4/4
Sensitivity	100%	75%	100%	100%
Specificity	85.9%	87.5%	85.9%	100%
Accuracy	88.3%	86.1%	89.7%	100%
Normalized values Ella				
Threshold (RFU)	120.8	88.4	81.9	59.61
Seronegative cohort	0/64	1/64	0/64	0/6
Seropositive cohort	13/13	7/8	23/23	4/4
Sensitivity	100%^#^	87.5%^##^	100%^#^	100%
Specificity	100%^#^	98.4%^##^	100%^#^	100%
Accuracy	100%	97.2%	100%	100%
Normalized standard ELISA [18]				
Sensitivity ELISA	100%	100%	92%	100%
Specificity ELISA	100%	100%	99.2%	100%

*Note:* Thresholds and number of positive Ella results in the respective cohort are shown for raw values and for normalized values, including the subtraction of the mean RFU when using BSA as a capture protein complementary to the target protein. Sensitivity, specificity, and accuracy could be increased by serum‐specific background normalization. For the comparison, diagnostic sensitivity and specificity of the in‐house standard ELISA (also including background normalization) are shown as assessed in a previous inter‐laboratory validation study [18]. ^#^refers to values assessed with known samples as well as within the diagnostic test cohort. ^##^refers to values assessed with known samples, not with the diagnostic test cohort. ^###^Limitation: Only tested on a reduced cohort.

### 3.3 Ella Shows Excellent Sensitivity and Specificity in Routine Diagnostic Testing

To validate the Ella platform as a new diagnostic tool, we screened a prospective test cohort of *n* = 37 patients with suspected AN and a typical clinical phenotype whose serum samples were sent to our laboratory for diagnostic assessment for anti‐contactin‐1 and anti‐neurofascin‐155. Here, 4/37 (10.81%) samples were detected as positive for anti‐neurofascin‐155, and 2/37 (5.41%) samples were detected as positive for anti‐contactin‐1. Validation with standard ELISA and CBA revealed that all hits were true positives (specificity: 100%). Normalized RFU values from the Ella platform correlated with normalized standard ELISA values (*r* = 0.85, *p* = 0.0025). Within the remaining *n* = 30 Ella negative samples, *n* = 1 sample showed positive results for anti‐Caspr‐1 ELISA/CBA (not assessed via Ella) and one sample showed weakly positive ELISA signal for anti‐neurofascin‐155 (OD = 0.481, threshold OD = 0.282; titer = 1:100), but negative results in the CBA validation assay. Thus, overall sensitivity—when defining double‐positivity in two standard assays as a gold standard—was 100%, in line with above calculated values using known positive and negative samples (see Table 2). Mean Ella RFU and ELISA OD values within the diagnostic test cohort are shown in Figure S2B.

### 3.4 Quality Assessment Against Standard ELISA

To quantitatively compare Ella to ELISA, titer analysis was performed for three seropositive sera per target antigen (excluding neurofascin‐186), showing consistency in dilution series and absolute titer compared to standard ELISA. Overall titers in the *n* = 9 samples correlated (*r* = 0.84, *p* = 0.0048, see Figure 1C). Individual titer values for all samples and titer dilution series exemplarily shown for anti‐contactin‐1 are provided in Figure S3. The titer correlation thus indicated a high degree of linearity of the test.

The quality of the assay was further analyzed by comparing intra‐ and inter‐assay CVs of paranodal targets to standard ELISA. Positive and negative results could be reproduced in every single intra‐assay and inter‐assay analysis, both in ELISA and in Ella, resulting in an overall intra‐assay and inter‐assay concordance of 100%. Normalized RFU intra‐assay variability was < 20% for both Ella and ELISA (*p* > 0.05, see Figure 1D). Inter‐assay variability was < 20% for both Ella assay and ELISA for 2/3 of the respective target antigens, without differences between the two assays (*p* > 0.05, see Figure 1E). Thus, both assays did not meet all high‐quality standards regarding exact RFU values, but showed comparable performance regarding variability, and excellent values for overall concordance of positive/negative results.

### 3.5 Low Frequency of Anti‐(Para)Nodal Antibodies in COVID‐19 Vaccinated Individuals

**FIGURE 2:**
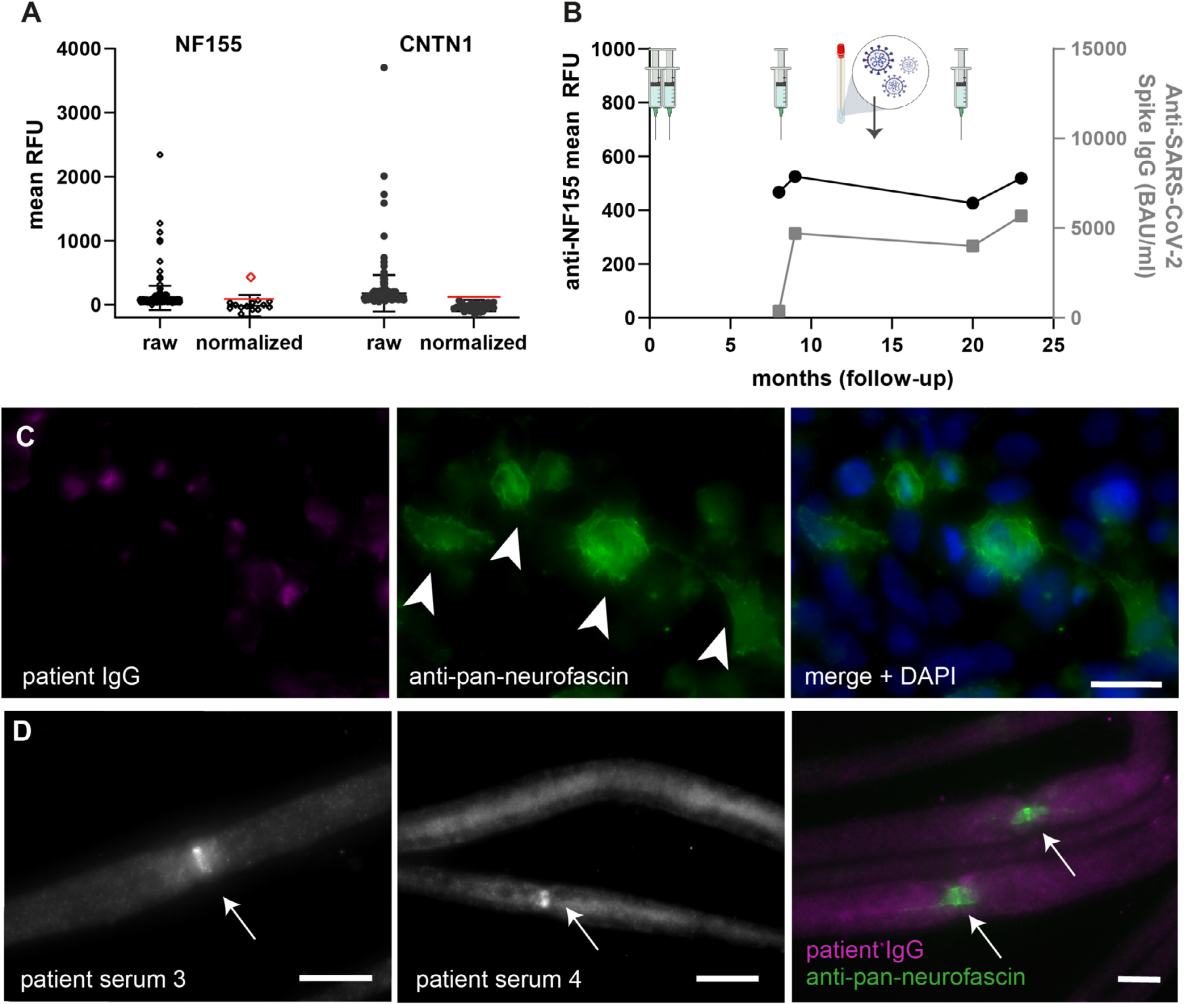
Screening for anti‐(para)nodal antibodies in SARS‐CoV‐2 vaccinated individuals and follow‐up. (A) Mean RFU values in the cohort of SARS‐CoV‐2‐vaccinated or infected individuals for anti‐NF155 (neurofascin‐155, left) and anti‐CNTN1 (Contactin‐1, right) antibodies, with means and standard deviation as error bars. Thresholds for positive results after normalization are indicated by the red line. Although many samples show high RFU in raw data, only one patient shows a positive result for NF155 after normalization (red square). (B) Clinical data including vaccination time points (syringe), Omicron infection (PCR), anti‐NF155 mean RFU values (left *y*‐axis, black), and anti‐SARS‐CoV‐2 spike IgG levels (right *y*‐axis, grey) of serum follow‐up samples over 25 months (*x*‐axis) in the Ella NF155 positive patient. (C) Serum binding assays with neurofascin‐transfected HEK293 cells show no specific binding of patient IgG diluted 1:100 (magenta, left) nor colocalization with anti‐pan‐neurofascin‐labeled, transfected cells (arrowheads, green). Overlay/merge is shown with DAPI staining on the right. Scale bar = 10 μm. (D) Murine teased fibers serum binding assays show IgG deposition at the nodal region (arrows) in follow‐up serum 3 (left) and 4 (middle) of the Ella NF155 positive patient, but not fully colocalizing with the paranodal NF155‐fraction when performing double staining with commercial anti‐pan‐neurofascin (green, right; patient IgG shown in magenta). Scale bar = 10 μm.

As a direct high‐throughput application of the new method, we tested a cohort of 280 individuals with a mean interval of three weeks after COVID‐19 vaccination or SARS‐CoV‐2 infection for antibodies against contactin‐1 and neurofascin‐155 using the Ella protocol. Neither mean raw RFU nor normalized RFU values correlated with anti‐SARS‐CoV‐2 spike IgG (*p* > 0.5 in Spearman correlation test). Although 5.4% (15/280) of sera showed increased raw mean RFU levels for anti‐neurofascin‐155, and 8.2% (23/280) for anti‐contactin‐1, only one positive result could be confirmed after applying serum‐specific background normalization (see Figure 2A). Thus, the frequency of anti‐neurofascin‐155 antibodies in the cohort was 0.36% (1/280) and 0% (0/280) for anti‐contactin‐1. The positive result was replicated thrice with similar outcomes. Titer analysis revealed an Ella anti‐neurofascin‐155 titer of 1:200.

For antibody validation in the positive individual, we conducted standard ELISA, immunofluorescence binding assays on murine nerve teased fibers, and CBAs. ELISA showed a negative result (OD 0.08, threshold 0.282), and CBA with neurofascin‐155‐transfected Human Embryonic Kidney cells did not show any specific binding of sample IgG to transfected cells at a dilution of 1:500 and 1:100 (see Figure 2C). Immunofluorescence binding assays on teased fibers revealed IgG deposition at the nodal region of the NoR in the two follow‐up sera of the neurofascin‐155 positive individual recruited after SARS‐CoV‐2 infection/fourfold COVID‐19 vaccination (patient serum 3 and 4), though not fully colocalizing with anti‐pan‐neurofascin double immunostaining (Figure 2D).

The Ella‐positive participant is a middle‐aged female individual vaccinated four times with Tozinameran (Pfizer‐BioNTech), without known autoimmune or malignant disease, and without SARS‐CoV‐2 infection upon inclusion, who had been tested positive for SARS‐CoV‐2 Omicron variant via PCR during the prospective follow‐up period (see Figure 2B). She did not report any neurological symptoms at any time point before or during follow‐up.

Longitudinal analysis using serum samples within a follow‐up period of 16 months showed constantly elevated normalized mean RFU values over time, independent of newly applied COVID‐19 vaccination or SARS‐CoV‐2 infection (see Figure 2B). No significant correlation was found between anti‐SARS‐CoV‐2 spike IgG levels and ODs (*p* = 0.54, *n* = 4).

In conclusion, positive anti‐neurofascin‐155 results were found at low frequency and with low titer, not validated by all three standard assays. Clinical data and longitudinal sample assessment did not indicate any SARS‐CoV‐2‐related fluctuation or symptoms.

## 4 Discussion

In this study, we established a sensitive and specific, highly automated ELISA platform for the detection of anti‐(para)nodal antibodies. Antibodies against contactin‐1, neurofascin‐155/186, and Caspr‐1 were identified with high accuracy compared to conventional methods, but with a 10‐fold reduction in hands‐on time, reduced sample volumes, and increased levels of automation.

Due to its excellent sensitivity, specificity, and reproducibility, the assay shows strong potential as a standardized diagnostic tool for detecting anti‐(para)nodal antibodies. Sensitivity and specificity of the Ella platform compare to 100% values of our standard in‐house ELISA system [18], and also to CBA, which has been reported to be 96%–100% sensitive and 97%–100% specific when performed on live cells, and 86%–91% sensitive and 99%–100% specific when performed on fixed cells [18, 20].

We present both analytical and clinical validation of the platform, benchmarked against our in‐house ELISA system. Intra‐assay coefficient of variation (CV) remained below 20%, confirming the high precision previously reported for the Ella platform [37]. Inter‐assay variation was precise for anti‐contactin‐1 and anti‐neurofascin‐155 as analytes, but showed slightly elevated variability for anti‐Caspr‐1. Here, cut‐off values were determined based on the mean RFU values of healthy controls, without applying dilution series or calibration curves, which may have limited sensitivity and contributed to increased variability. Nevertheless, this approach aligns with current standard protocols for anti‐(para)nodal ELISA [18]. Future implementation of human monoclonal, epitope‐specific anti‐(para)nodal antibodies with defined concentrations may enhance assay precision and potentially increase sensitivity to the nanoscale, similar to other Ella‐validated biomarkers [21, 24]. Further, larger multicenter studies will need to assess interlaboratory reproducibility of results, robustness in different settings, and clinical validation, also compared to other gold‐standard methods like CBA.

We show that serum‐specific background normalization is crucial to achieve high sensitivity and specificity, as previously reported for standard ELISA [38], especially when not using calibration curves. Here, we propose to perform normalization in a second validation step, serving a dual purpose: First, we replicate positive results, and second, we save valuable cartridge space by just repeating conspicuous results (which was the case in 5%–14% of tested sera). In the future, serum‐specific background correction should be routinely integrated into both standard and automated ELISA workflows to improve diagnostic accuracy.

A potential disadvantage of the new platform is the higher cost of single‐use cartridges compared to conventional ELISA plates. However, these expenses may be offset by reduced personnel costs due to automation, significantly lower hands‐on time, and lower consumption of costly protein reagents due to minimized sample volume.

In summary, the present work shows that an automated, high‐throughput ELISA platform can reliably detect anti‐(para)nodal antibodies with high accuracy and reproducibility, paving the way for standardized, large‐scale serological diagnostics in immune‐mediated neuropathies.

In a cohort of 280 individuals after SARS‐CoV‐2 infection or vaccination, only a very low frequency of anti‐neurofascin‐155 antibodies was observed, with no clinical evidence of neuropathic symptoms. The low‐titer anti‐neurofascin‐155 response detected by the Ella platform in one individual was not confirmed by standard ELISA or CBA, and teased fiber immunostaining showed local IgG reactivity not colocalizing at the paranode, thus indicating a false positive result. Further, the clinical lack of symptoms argues against a pathogenic relevance of the test result.

The immunological relevance of autoantibodies following SARS‐CoV‐2 infection or COVID‐19 vaccination remains subject to ongoing investigation. COVID‐19 appears to be a potential risk factor for developing ANA‐associated autoimmune conditions [39]. Furthermore, the spectrum of neurological complications of COVID‐19 has been constantly growing, including autoimmune conditions such as brainstem encephalitis and myelitis [40, 41]. Although associated with specific antibodies like Caspr‐2 or anti‐ganglioside antibodies in some cases [42, 43], the targets of the autoimmune response after SARS‐CoV‐2 infection largely remain unknown [41]. IgG hippocampus, cerebellar, and brainstem reactivity might be linked to post‐acute COVID‐19 sequelae and also relate to cognitive decline, but detailed pathophysiological studies and evidence are still lacking [44, 45].

COVID‐19 vaccination can reduce the risk of SARS‐CoV‐2 infection, severe COVID‐19 disease course, and post‐acute COVID‐19 sequelae, which might indirectly lower the risk for autoimmune complications [46, 47, 48]. Still, studies also report autoimmune phenomena after COVID‐19 vaccination, although discussed controversially [49, 50, 51]. In the PNS, high IgG reactivity against peripheral nerve structures, including the NoR, has been described in patients with neurological complications, including sensory symptoms and neuropathic pain after mRNA vaccination, with neurofilaments and DFS‐70 as potential antigens [52]. However, mRNA vaccines appear to have a lower propensity for triggering autoimmune responses than vector‐based vaccines, and anti‐(para)nodal antibodies do not occur after vaccination in other neuroimmunologic diseases like Multiple Sclerosis [51, 53]. Potential antibody responses after vaccination in healthy individuals have not been thoroughly studied.

GBS, an inflammatory neuropathy very similar to AN, is often preceded by infections or vaccinations, including SARS‐CoV‐2 [6, 7, 54]. Unlike *Campylobacter jejuni* , whose ganglioside‐like proteins can induce cross‐reactive autoimmunity [55], SARS‐CoV‐2 lacks confirmed molecular mimicry mechanisms thus far [7]. Consequently, the relevance of SARS‐CoV‐2 in promoting autoantibody generation remains uncertain.

Case reports have reported AN and anti‐(para)nodal antibodies in temporal association to SARS‐CoV‐2 infection or vaccination [11, 12, 13, 43]. Although structural similarity between anti‐(para)nodal antibodies and SARS‐CoV‐2 has not been demonstrated, the adhesion protein contactin‐1 was found to interact with the SARS‐CoV‐2 receptor binding domain, being upregulated in COVID‐19 and possibly serving as a co‐receptor [14]. This raises the hypothesis that contactin‐1 may serve as a target for autoantibody generation as part of a natural defense mechanism against SARS‐CoV‐2 infection. Still, we did not identify any antibodies against anti‐contactin‐1 in our postinfection or postvaccination cohort, arguing against contactin‐1 immunity as a frequent event. Furthermore, we did identify anti‐neurofascin‐155 antibodies in only one individual, at a low titer and without clinical implications.

Overall, our findings support the safety of mRNA‐based COVID‐19 vaccination concerning autoimmune neuropathies. Nevertheless, broader population‐based studies with long‐term clinical monitoring are needed due to the rarity of these antibody responses.

In conclusion, we offer a valuable tool to enhance clinical diagnostics and decision‐making as well as understanding disease mechanisms in inflammatory neuropathies.

## Funding

The study was funded by a research grant from the German Muscle Disorder Society (Deutsche Gesellschaft für Muskelkranke, DGM e.V., AP1/1) to L.A. and K.D. The study was further supported by the German Federal Ministry of Education and Research (BMBF) as part of the Network University Medicine (NUM): “NaFoUniMedCovid19” Grant No: 01KX2021, Project: “Bundesweites Forschungsnetz Angewandte Surveillance und Testung” (B‐FAST) by the BMBF Network of University Medicine 2.0: “NUM 2.0,” Grant No. 01KX2121, Project: COVIM 2.0, and by the Free State of Bavaria with COVID‐19‐research funds provided to the Julius‐Maximilians‐Universität Würzburg, Germany.

## Supporting information


**Figure S1:** ELISA for digoxigenin labeling validation. Graphs show the mean ELISA optical density (OD_450_) values and standard deviation for anti‐contactin‐1, anti‐Caspr‐1, and anti‐neurofascin‐155 antibodies, when evaluated with healthy control (HC) serum, patient serum, commercial antibodies against the respective target (com.ab), and an HRP‐conjugated anti‐digoxigenin secondary antibody (dig.ab). As coating proteins, we used either digoxigenin‐conjugated protein (light grey) or the original protein (dark grey) at equal dilutions. Mean ODs are comparably high in patient and commercial samples, and the anti‐digoxigenin control is positive when using the conjugated protein, validating labeling success.

**Figure S2:** Mean RFU raw values for Ella validation and ROC curves illustrating the diagnostic performance of the test (A) Mean RFU raw values in healthy controls (HC), seronegative (SN) and seropositive (SP) patients, using neurofascin‐155 (NF155), Contactin‐1 (CNTN1), Caspr‐1, and neurofascin‐186 (NF186) as target protein. (B) Charts show mean RFU values assessed by Ella and OD values assessed by standard ELISA within the prospective diagnostic test cohort of *n* = 37 samples for Contactin‐1 (CNTN1) and neurofascin‐155 (NF155). (C) The dotted line represents the ROC curve showing the trade‐off between sensitivity (true positive rate) and 100% specificity (false positive rate) for mean raw RFU values with neurofascin‐155 (NF155, left), Contactin‐1 (CNTN1, middle), and Caspr‐1 (right) as a target. The area under the curve (AUC) quantifies the overall accuracy of Ella, with an AUC of 1.0 indicating perfect classification and 0.5 representing no discriminative power. The dashed diagonal red line corresponds to the performance of a random classifier (AUC = 0.5) as a reference. (D) Similar to (C), but showing normalized RFU values for the respective target antigen.

**Figure S3:** Titer comparison and anti‐contactin‐1 titer dilution series (A) Table showing titers determined by the Ella platform and by standard ELISA, for three patients per target antigen. (B) Dilution series of three anti‐contactin‐1 seropositive samples in Ella are shown. The red line indicates the threshold for a positive result (normalized).

**Table S1:** Descriptive cohort data. Abbreviations: AN = autoimmune nodopathy, Caspr‐1 = Contactin‐1‐associated protein 1, CIDP = Chronic Inflammatory Demyelinating Polyradiculoneuropathy, CNTN1 = Contactin1, n/a = not assessed, f = female, m = male, NF155 = Neurofascin‐155, PanNF = Pan‐Neurofascin. For age and interval to last SARS‐CoV‐2 immunizing event, Inter Quartile Range is given in brackets.

## Data Availability

The data that support the findings of this study are available from the corresponding author upon reasonable request.
