# Supplementary figures and images for: A Next‐Generation ELISA for the Detection of Anti‐(Para)Nodal Antibodies in Autoimmune Nodopathy and COVID‐19 Vaccinated Individuals
Source: J Peripher Nerv Syst. 2026 Mar 29;31(2):e70117. doi: 10.1111/jns.70117 (PMC13033911; doi:10.1111/jns.70117)

**contactin-1**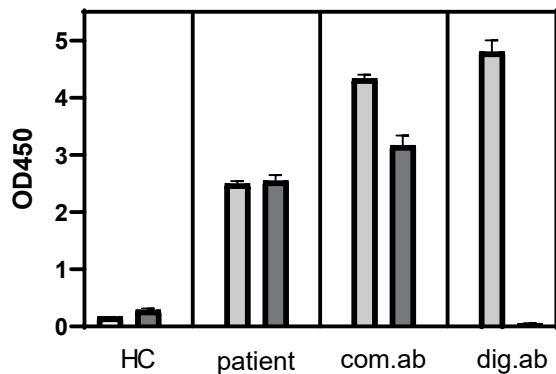**Caspr-1**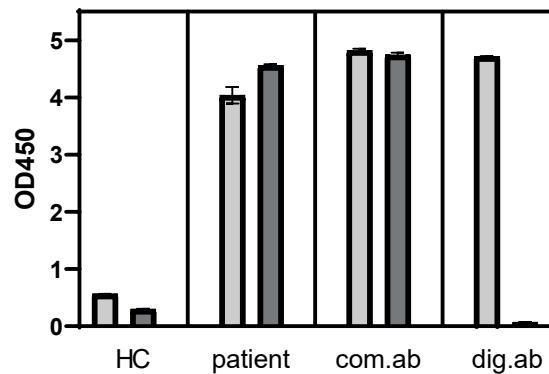**neurofascin-155**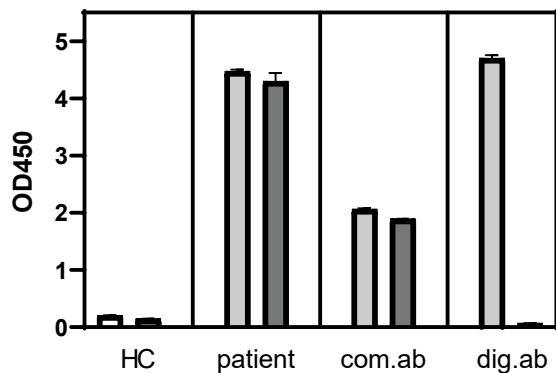**neurofascin-186**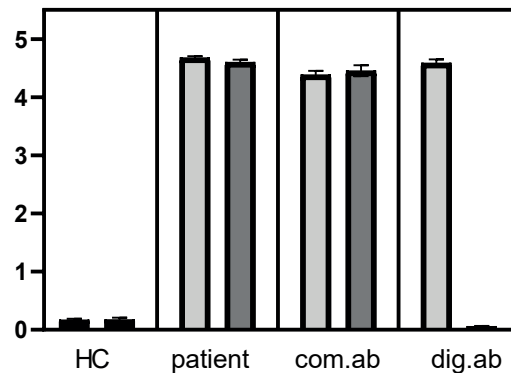

□ digoxigenin-conjugated protein    ■ original protein

Supplement: Supplementary file 1 — Figure S1: ELISA for digoxigenin labeling validation. Graphs show the mean ELISA optical density (OD450) values and standard deviation for anti‐contactin‐1, anti‐Caspr‐1, and anti‐neurofascin‐155 antibodies, when evaluated with healthy control (HC) serum, patient serum, commercial antibodies against the respective target (com.ab), and an HRP‐conjugated anti‐digoxigenin secondary antibody (dig.ab). As coating proteins, we used either digoxigenin‐conjugated protein (light grey) or the original protein (dark grey) at equal dilutions. Mean ODs are comparably high in patient and commercial samples, and the anti‐digoxigenin control is positive when using the conjugated protein, validating labeling success. [file JNS-31-0-s003.pdf]

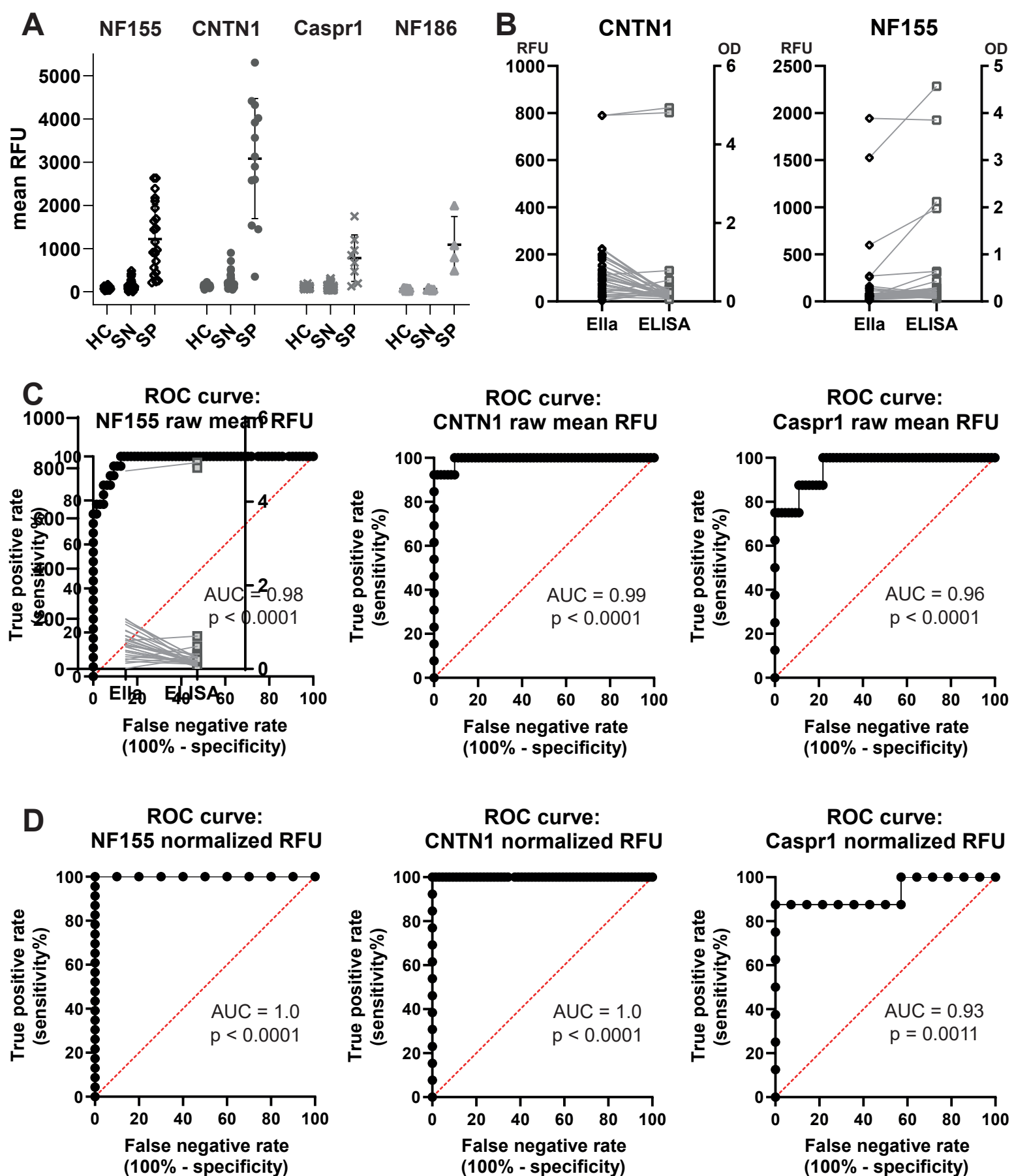

Supplement: Supplementary file 2 — Figure S2: Mean RFU raw values for Ella validation and ROC curves illustrating the diagnostic performance of the test (A) Mean RFU raw values in healthy controls (HC), seronegative (SN) and seropositive (SP) patients, using neurofascin‐155 (NF155), Contactin‐1 (CNTN1), Caspr‐1, and neurofascin‐186 (NF186) as target protein. (B) Charts show mean RFU values assessed by Ella and OD values assessed by standard ELISA within the prospective diagnostic test cohort of n = 37 samples for Contactin‐1 (CNTN1) and neurofascin‐155 (NF155). (C) The dotted line represents the ROC curve showing the trade‐off between sensitivity (true positive rate) and 100% specificity (false positive rate) for mean raw RFU values with neurofascin‐155 (NF155, left), Contactin‐1 (CNTN1, middle), and Caspr‐1 (right) as a target. The area under the curve (AUC) quantifies the overall accuracy of Ella, with an AUC of 1.0 indicating perfect classification and 0.5 representing no discriminative power. The dashed diagonal red line corresponds to the performance of a random classifier (AUC = 0.5) as a reference. (D) Similar to (C), but showing normalized RFU values for the respective target antigen. [file JNS-31-0-s004.pdf]
