# Supplementary material for: A Next‐Generation ELISA for the Detection of Anti‐(Para)Nodal Antibodies in Autoimmune Nodopathy and COVID‐19 Vaccinated Individuals
Source: J Peripher Nerv Syst. 2026 Mar 29;31(2):e70117. doi: 10.1111/jns.70117 (PMC13033911; doi:10.1111/jns.70117)

A ELISA and Ella Titers

antigen	patient	ELISA	Ella
Caspr-1	patient 1	1:2000	1:2000
	patient 2	1:8000	1:12000
	patient 3	1:300	1:1500
NF155	patient 4	1:500	1:500
	patient 5	1:1000	1:1000
	patient 6	1:2000	1:3000
CNTN1	patient 7	1:2000	1:2000
	patient 8	1:500	1:400
	patient 9	1:30000	1:12800

B

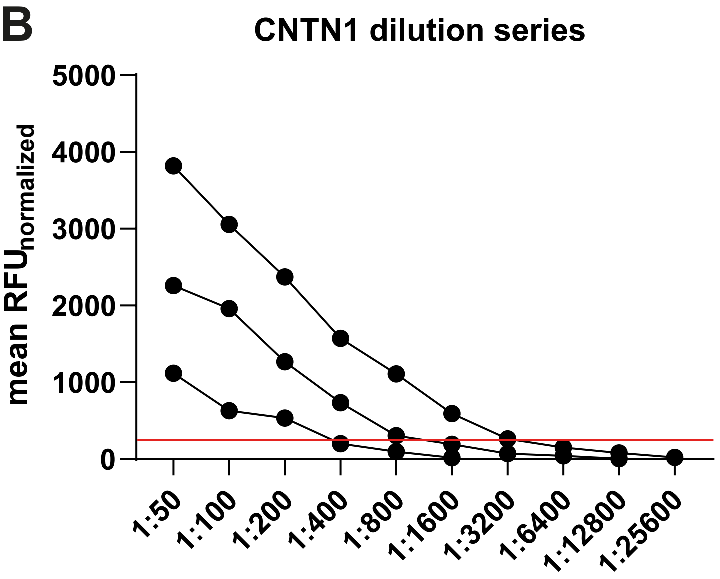

Supplement: Supplementary file 3 — Figure S3: Titer comparison and anti‐contactin‐1 titer dilution series (A) Table showing titers determined by the Ella platform and by standard ELISA, for three patients per target antigen. (B) Dilution series of three anti‐contactin‐1 seropositive samples in Ella are shown. The red line indicates the threshold for a positive result (normalized). [file JNS-31-0-s002.pdf]
