# Supplementary material for: A Next‐Generation ELISA for the Detection of Anti‐(Para)Nodal Antibodies in Autoimmune Nodopathy and COVID‐19 Vaccinated Individuals
Source: J Peripher Nerv Syst. 2026 Mar 29;31(2):e70117. doi: 10.1111/jns.70117 (PMC13033911; doi:10.1111/jns.70117)
Supplement: Supplementary file 4 — Table S1: Descriptive cohort data. Abbreviations: AN = autoimmune nodopathy, Caspr‐1 = Contactin‐1‐associated protein 1, CIDP = Chronic Inflammatory Demyelinating Polyradiculoneuropathy, CNTN1 = Contactin1, n/a = not assessed, f = female, m = male, NF155 = Neurofascin‐155, PanNF = Pan‐Neurofascin. For age and interval to last SARS‐CoV‐2 immunizing event, Inter Quartile Range is given in brackets. [file JNS-31-0-s001.pdf]

Supporting Table 1: Descriptive cohort data. Abbreviations: AN = autoimmune nodopathy, Caspr-1 = Contactin-1 associated protein 1, CIDP = Chronic Inflammatory Demyelinating Polyradiculoneuropathy, CNTN1 = Contactin1, n/a = not assessed, f= female, m = male, NF155 = Neurofascin-155, PanNF = Pan-Neurofascin. For age and interval to last SARS-CoV-2 immunizing event, Inter Quartile Range is given in brackets.

**cohort**	**number of participants**	**age (range) in years**	**sex**	**last COVID-19 vaccination**	**number of COVID-19 vaccinations**	**SARS-CoV-2 infection (number)**	**Interval to last SARS-CoV-2 immunising event (vaccination / infection) in days**
Healthy controls	30	59 (47 – 84)	11 f<br>19 m	none	0	0	n/a
Seronegative acute-onset CIDP	64	58 (12 – 84)	21 f<br>43 m	n/a	n/a	n/a	n/a
Seropositive AN	44 (13 CNTN1, 8 Caspr-1, 11 NF155, 12 PanNF)	57 (18 – 83)	10 f<br>34 m	n/a	n/a	n/a	n/a
Prospecitve test cohort	37	63 (24 – 88)	14 f<br>23 m	n/a	n/a	n/a	n/a
CoVacSer study	280	46 (18 – 76)	40 m<br>240 f	BNT162b2mRNA Original (Comirnaty Original, BioNTech/Pfizer, Mainz/Germany, New York/USA): n= 187<br><br>BNT162b2mRNA BA.1 BA.1: n = 6<br><br>BNT162b2mRNA Omicron BA.4-5: n = 51<br><br>mRNA-1273 (Spikevax, Moderna, Cambridge/USA): n = 36	2 (n = 4)<br>3 (n = 103)<br>4 (n = 172)<br>5 (n = 1)	0 (n = 85)<br>1 (n = 151)<br>2 (n = 42)<br>3 (n = 1)	22 (2 – 100)
